# Value of peri-operative chemotherapy in patients with CINSARC high-risk localized grade 1 or 2 soft tissue sarcoma: study protocol of the target selection phase III CHIC-STS trial

**DOI:** 10.1186/s12885-020-07207-3

**Published:** 2020-07-31

**Authors:** Thomas Filleron, Sophie Le Guellec, Christine Chevreau, Bastien Cabarrou, Tom Lesluyes, Sabrina Lodin, Angélique Massoubre, Muriel Mounier, Muriel Poublanc, Frédéric Chibon, Thibaud Valentin

**Affiliations:** 1 Biostatistics Unit, Institut Claudius Regaud, IUCT-O, 1 Avenue Irène Joliot Curie, 31059 Toulouse Cedex, France; 2 Department of Pathology, Institut Claudius Regaud, IUCT-O, 1 Avenue Irène Joliot Curie, 31059 Toulouse, France; 3 INSERM U1037, Cancer Research Center of Toulouse (CRCT), Toulouse, France; 4 Department of Medical Oncology, Institut Claudius Regaud IUCT-O, 1 Avenue Irène Joliot Curie, 31059 Toulouse, France; 5 Clinical Trials Office, Institut Claudius Regaud, IUCT-O, 1 Avenue Irène Joliot Curie, 31059 Toulouse, France

**Keywords:** Soft tissue sarcoma, CINSARC signature, Chemotherapy, Target selection design

## Abstract

**Background:**

The value of chemotherapy in soft tissue sarcoma (STS) remains controversial. Several expert teams consider that chemotherapy provides a survival advantage and should be proposed in high-risk (HR) patients. However, the lack of accuracy in identifying HR patients with conventional risk factors (large, deep, FNCLCC grade 3, extremity STS) is an issue that cannot be neglected. For example, while the FNCLCC grading system is a powerful tool, it has several limitations. CINSARC, a 67-gene signature, has proved to be an additional independent factor for predicting metastatic spread and outperforms histological grade. Regardless of FNCLCC grade, CINSARC stratifies patients into two separate prognostic groups: one with an excellent prognosis (low-risk (LR) CINSARC) and the other with a worse outcome (HR-CINSARC) in terms of metastatic relapse. Here we evaluate the role of chemotherapy in grade 1–2 STS patients with HR-CINSARC and assess the prognostic value of CINSARC in patients treated with standard of care.

**Methods:**

CHIC is a parallel, randomized, open-label, multicenter study evaluating the effect on metastasis-free survival of adding perioperative chemotherapy to standard of care in patients with grade ½ STS sarcoma defined as HR by CINSARC. In this target selection design, 600 patients will be screened with CINSARC to randomize 250 HR-CINSARC patients between standard of care and standard of care plus chemotherapy (4 cycles of 3 weeks of intravenous chemotherapy with doxorubicin in combination with dacarbazine or ifosfamide according to histologic subtype). LR-CINSARC patients will be treated by standard of care according to the investigator. The primary endpoint is metastasis-free survival. Secondary endpoints include overall survival, disease-free survival and safety. Furthermore, the prognostic value of CINSARC will be evaluated by comparing LR-CINSARC patients to HR-CINSARC patients randomized in standard of care.

**Discussion:**

CHIC is a prospective randomized phase III trial designed to comprehensively evaluate the benefit of chemotherapy in HR-CINSARC patients and to prospectively validate the prognostic value of CINSARC in grade ½ STS sarcoma patients.

**Trial registration:**

ClinicalTrials.gov identifier: NCT04307277 Date of registration: 13 March 2020

## Background

With an incidence of 4–5 cases / 1,000,000, soft tissue sarcomas (STS) are a group of rare heterogeneous tumors that develop from connective tissue cells. Their therapeutic management is based on multidisciplinary discussion at every step, starting with the need for a pre-therapeutic diagnostic biopsy. The key point in treating patients with localized STS is a large surgical resection to obtain a microscopically negative margin and remove all tumor cells. The quality of surgery remains the main risk factor for metastatic relapse. Neo-adjuvant or adjuvant treatments (radiotherapy and/or chemotherapy) are discussed case by case in a multidisciplinary meeting, depending on the presence of other prognostic factors such as histologic subtype, tumor size and FNCLCC (Fédération Nationale des Centres de Lutte Contre le Cancer) grade.

In recent decades, the role of perioperative (mainly adjuvant) chemotherapy in patients with localized STS has been extensively investigated. Currently, even after multiple randomized studies supplemented by two meta-analyses [1, 2] the survival benefit of chemotherapy in all patients with localized STS remains debated. In current guidelines [3], chemotherapy may be proposed as an option in HR patients but it is not part of standard of care. This uncertainty regarding the real impact of chemotherapy can be explained by the diversity of the randomized studies in terms of drug type, tumor grade, location, and quality of surgery, each of these factors being essential. Based on previous published studies, many expert teams currently consider that chemotherapy provides a survival advantage and should be proposed in HR patients [4]. This HR subgroup includes patients with large, deep, high FNCLCC grade, and extremity STS [5]. One study specifically included these patients yet showed an improvement in survival [6]. However, the debate concerning the value of chemotherapy in localized STS is currently in deadlock, mirroring the difficulty of accurately identifying HR patients by using conventional factors, and more specifically the FNCLCC grade. This grading system, which splits STS into three grades (1, 2 or 3), remains the best predictor of metastatic relapse available in localized sarcoma [5, 7]. Despite its consensual use in determining the optimal therapeutic strategy of patients with STS [8], it has several limitations. It is not applicable in all pathological subtypes, it shows variable reproducibility between pathologists, and it is difficult to use with tumor microbiopsies, i.e. the gold standard for diagnosis. Above all, however, FNCLCC grade has a poorly informative prognostic value for grade 2, which represents about 40% of all STS. Therefore, the CINSARC signature, which is especially informative in patients with intermediate-grade tumors, could be a way out of this “never-ending story”.

### CINSARC signature

In 2010, Chibon et al. identified a 67-gene expression signature, CINSARC (Complexity Index in SARComas), and validated it as an independent prognostic factor in several STS histotypes [9]. They also demonstrated that it outperformed histologic grade and was able to split STS into two separate prognostic groups, regardless of FNCLCC grade. CINSARC is especially informative in patients with intermediate-grade tumors, i.e. about 40% of all STS, for whom FNCLCC grade is poorly informative. In this population of uncertain prognosis, CINSARC identified around 50% of patients as having a high risk of metastatic relapse [10]. These patients have a more than 60% risk of metastatic relapse, as compared to 20% for LR-CINSARC patients. These rates are similar to what is reported in grade 3 tumors, confirming that CINSARC identifies HR patients regardless of FNCLCC grade.

Until recently, the main clinical hurdle for using CINSARC was technical, since it was performed on frozen tumors analyzed by microarrays, features incompatible with usual routine settings. It was first used with formalin-fixed, paraffin-embedded (FFPE) RNA sequencing [11], and is now fully evaluable in FFPE tumors using Nanostring technology [12]. Nanostring is a recent technology with two major advantages: only needs small quantities of tumoral DNA (feasible in microbiopsies), and perfect reproducibility between different machines and centers. Optimization of CINSARC in FFPE samples using NanoString (named NanoCind®, patent number EP18305190.3) is the final step for considering the use of CINSARC on diagnostic FFPE microbiopsies in routine settings for patients with localized STS, and it can now be used to guide treatment [13].

### Research hypothesis

Chemotherapy is currently not part of the treatment of patients with grade 1/2 (G1/2) localized STS, because they are not considered as being at high risk of relapse according to the classical factors. Since chemotherapy is considered beneficial in HR-STS patients, we hypothesize that CINSARC could be the key to identifying these patients differently and proving once and for all the benefit of chemotherapy in G1/2 STS. Our hypothesis is that in G1/2 STS patients considered as HR according to the CINSARC signature, the addition of four cycles of perioperative doxorubicin-based chemotherapy could improve metastasis-free survival (MFS) as compared with standard management.

## Methods and design

### Trial objectives

#### Primary objective

The primary objective of this study is to demonstrate whether adding four cycles of perioperative doxorubicin-based chemotherapy (combination of doxorubicin and ifosfamide/dacarbazine) improves MFS as compared with standard management in patients with resectable FNCLCC G1/2 STS, considered as HR according to CINSARC.

#### Secondary objectives

Secondary objectives include the comparison of the two therapeutic strategies in HR-CINSARC patients with G1/2 resectable STS in terms of disease-free survival (DFS), overall survival (OS) and safety profile. Moreover, the prognostic value of CINSARC in G1/2 STS treated by standard treatment is evaluated prospectively.

### Trial design

This is a phase III, multicenter, randomized open-label comparative study that has been designed to demonstrate whether adding four cycles of perioperative doxorubicin-based chemotherapy (doxorubicin and ifosfamide or dacarbazine) improves MFS as compared with standard management in patients with resectable FNCLCC G1/2 STS, considered as HR according to CINSARC (ClinicalTrials.gov Identifier: NCT04307277).

**Fig. 1:**
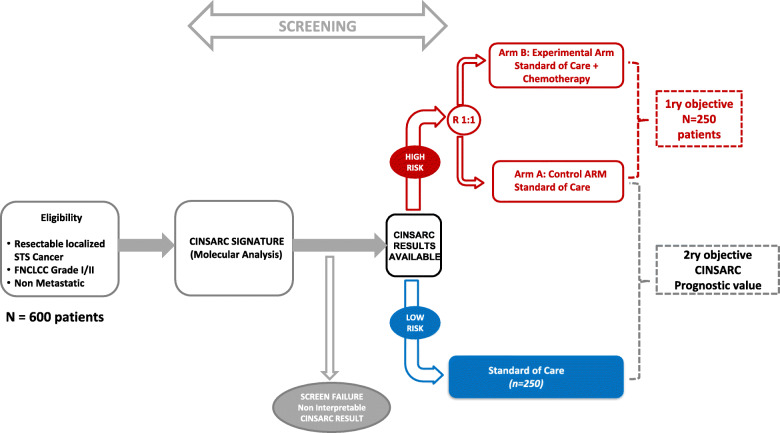
Study design of CHIC trial

After providing written informed consent, patients considered as eligible for the CHIC-STS study by the investigator will be enrolled and molecular screening will be performed to determine their classification according to CINSARC (LR or HR). To evaluate both the effect of chemotherapy in the subgroup of patients with an HR-CINSARC signature and the prognostic value of the CINSARC signature, this study is based on a target selection design [14]. Patients for whom tumor material has been qualified and CINSARC classification has been determined will be definitively enrolled according to their CINSARC classification results (Fig. 1).

- Patients classified as HR by CINSARC signature (HR-CINSARC) will be randomized between standard of care (surgical excision +/− external radiotherapy) and the experimental arm (standard of care with chemotherapy)
- Patients classified as LR by CINSARC signature (LR-CINSARC) will be treated at the discretion of the clinicians and data will be collected prospectively.

### Clinical study endpoints

#### Primary endpoint

The primary endpoint is MFS, which is defined as the delay between randomization and the onset of metastatic disease or death from any cause [15]. In the event of initial locoregional relapse, patients will be censored at the date of onset. Patients still alive at the time of analysis (including lost to follow-up) without onset of metastatic disease will be censored at the last disease assessment date.

#### Secondary endpoints

DFS is defined as the delay between randomization and first relapse (local, regional, or distant) or death from any cause. Patients still alive at the time of analysis (including lost to follow-up) without relapse will be censored at the last disease assessment date.

OS is defined as the delay between randomization and death from any cause. Patients still alive at the time of analysis (including lost to follow-up) will be censored at the last known date alive.

Safety will be assessed by the toxicity grading system of the National Cancer Institute (NCI-CTCAE v5.0).

### Screening and randomization

The target population is patients with resectable FNCLCC G1/2 STS. After giving their written informed consent, patients fulfilling all inclusion and non-inclusion criteria (Table 1) will be enrolled in the study and molecular screening will be performed. After inclusion, an archived FFPE tumor sample of sufficient quantity will be sent to one of the four identified CINSARC signature platforms. Results of CINSARC analysis classifying patients as HR or LR-CINSARC will be provided by email to the investigator. After fulfilling additional inclusion criteria (HR-CINSARC signature, external radiotherapy not initiated before randomization (if applicable), full details in Table 1)), HR-CINSARC patients will be randomized by the sponsor to one of the two arms in a 1:1 ratio. Randomization will be stratified on the following factors: center, FNCLCC grade (grade 1 vs grade 2), tumor size (< 5 cm vs ≥5 cm), timing of chemotherapy envisaged (neo-adjuvant vs adjuvant) and histology (undifferentiated pleomorphic sarcoma vs dedifferentiated liposarcoma vs leiomyosarcoma vs synovial sarcoma vs other). A dynamic randomization procedure by minimization will be used. Randomization will be performed centrally by the IUCT-O clinical trials office using the TENALEA Clinical Trial Data Management System (online secure internet).

**Table 1:** Inclusion and non-inclusion criteria

Inclusion Criteria	Non Inclusion criteria
- The regional platform will transmit the CINSARC status to the sponsor and will be provided to the investigator.According to FNCLCC grading system, grade 2 and grade 1 tumors - Resectable and localized disease after appropriate extension work-up (including at least a chest-CT) - Available archived FFPE tumor sample in sufficient quantity to allow CINSARC qualification - Age ≥ 18 years - Eastern Cooperative Oncology Group (ECOG) performance status ≤2 - Life expectancy of at least 12 weeks after the start of the treatment - Women should be post-menopaused or willing to accept the use an effective contraceptive regimen during the treatment period and at least 12 months after the end of the treatment period. All non-menopaused women should have a negative pregnancy test within 72 h prior to registration. Men should accept to use an effective contraception during treatment period and at least 3 months (Ifosfamide treatment) or 6 months (Dacarbazine treatment) after the end of the study treatment - Signed written informed consent - Patient affiliated to a Social Health Insurance in France **Additional criteria: Randomized Part** - High-risk CINSARC signature -External radiotherapy not initiated before randomization (if applicable). -Acceptable hematologic function (within 72 h prior randomization): Absolute neutrophil count (ANC) ≥ 1.5 G/L, Platelet count ≥100 G/L and Hemoglobin > 9 g/dL -Acceptable renal function within 72 h prior randomization: Serum creatinine ≤1.5 x ULN or calculated creatinine clearance ≥60 mL/min (by the Cockcroft and Gault formula) -Acceptable liver function: Bilirubin ≤1.5 x upper limit of normal (ULN), AST (SGOT) and ALT (SGPT) ≤ 2.5 x ULN -Normal LVEF (> 50%) measured by echocardiography or isotopic ventriculography	- Soft-tissue sarcoma with the following histological subtypes: well-differentiated liposarcomas, alveolar soft-part sarcoma, dermatofibrosarcoma protuberans, clear-cell sarcoma, epithelioid sarcoma, alveolar or embryonal rhabdomyosarcoma - Primitive cutaneous, retroperitoneal, uterus or visceral STS - Metastatic disease - Previous or ongoing treatment for the sarcoma (with the exception of a surgery for diagnosis intend) - Contra-indication for Doxorubicin, Ifosfamide and Dacarbazine treatments - Prior therapy with ifosfamide or cyclophosphamide or other nitrogen mustards, and prior therapy with anthracyclines - Prior mediastinal/cardiac radiotherapy - History or presence of clinically relevant cardiovascular abnormalities such as uncontrolled hypertension, congestive heart failure NYHA classification of 3, unstable angina or poorly controlled arrhythmia, myocardial infarction within 6 months prior to study entry - Prior or concurrent malignant disease diagnosed or treated in the last 2 years except for adequately treated in situ carcinoma of the cervix, basal or squamous skin cell carcinoma, or in situ transitional bladder cell carcinoma - Active, uncontrolled bacterial, viral, or fungal infections, requiring systemic therapy - Known infection with HIV, hepatitis B, or hepatitis C - Women who are breastfeeding, pregnant or who plan to become pregnant while in the trial - Concomitant disease or condition that could interfere with the conduct of the study, or that would, in the opinion of the investigator, pose an unacceptable risk to the subject in this study - Patient who has forfeited his/her freedom by administrative or legal award or who is under legal protection (curatorship and guardianship, protection of justice) - Patient unable to comply with the protocol for any reason.

LR-CINSARC patients will be included and treated at the discretion of the clinicians and data will be collected prospectively.

### CINSARC analysis

For each patient, a representative sample formalin-fixed paraffin-embedded (FFPE) tumor block from sarcoma tumor surgical resection or biopsy specimen (archived tissue) will be selected for molecular screening. Quality control of samples will be assessed by the investigational site pathologist and samples with less than 50% of tumor cells will be considered as non-eligible for CINSARC analysis (screen failure). Tumor samples passing quality control will be addressed to one of the four regional platforms.

CINSARC analysis will be performed using Nanostring technology [16, 17]. Briefly, unique multiplexed probes are made with two sequence-specific probes complementary to a 100-base target region per target mRNA. The capture probe comprises a target-specific oligonucleotide coupled with a short sequence linked to biotin. The reporter probe consists of a second 50mer target-specific oligonucleotide linked to a unique chain of dye-labeled segments for detection by the system. Our nCounter code set, named NanoCind®, includes a panel of 75 probes, including 67 distinct test probes derived from 67 distinct genes and eight housekeeping genes for normalization purposes. Probe sets were designed and synthesized by NanoString. Additional information concerning probes can be found in the patent, which is filed under the number EP18305190.3. This raw data can be uploaded to a web interface developed for this study and hosted by the Institute Claudius Regaud. The interface will calculate the CINSARC status: “LR” or “HR”. The regional platform will transmit the CINSARC status to the sponsor, who will send it in turn to the investigator.

### Treatment

#### Standard of care

HR-CINSARC patients randomized to the control arm and LR-CINSARC patients will be treated according to standard of care. Surgery will be performed according to standard guidelines by a surgeon in one of the investigational participating centers. If indicated, patients may be treated by external radiotherapy before or after excision surgery (if applicable) according to each participating center’s procedures and treatment guidelines.

#### Experimental treatment

Patients classified as HR-CINSARC randomized to the experimental treatment will be treated by standard of care with the addition of chemotherapy. They will receive 4 cycles of 3 weeks of intravenous chemotherapy with doxorubicin (20 mg/m2 per day [day 1, 2 and 3] every 3 week) in combination with dacarbazine (300 mg/m2 per day [day 1, 2 and 3] every 3 week)) for leiomyosarcoma or ifosfamide (3 g/m2 per day [day 1, 2 and 3] every 3 week) for other histologic subtypes.

### Follow-up

HR-CINSARC patients (Arm A or B) will be followed from the date of randomization, every 4 months the two first years and then every 6 months the next 3 years for a maximum of 5 years. According to treatment guidelines, assessments during this 5-year follow-up period will include at least clinical examinations of the tumor primary site and overall assessment/metastatic relapse assessment by CT scan. In the event of relapse, patients will be followed only for survival status until 5 years post-randomization.

Patients defined as LR-CINSARC will be followed up according to local practice and relevant prospective data (baseline characteristics, treatment, carcinologic event and survival status) will be collected for 5 years from the date of registration in the prospective cohort.

### Main statistical analysis

#### HR-CINSARC patients: randomized part

In G1/2 HR-CINSARC, the 3-year MFS was estimated around 60% [9, 10]. The main objective is to increase 3-year MFS from 60 to 75%, which corresponds to detecting a hazard ratio of 0.56. A total of 101 events is necessary for 80% power to detect this difference if it is true using a one–sided Logrank test at the 2.5% level of significance and a 1:1 randomization. Based on an estimated accrual rate of approximately 8 patients per month for the randomization of 250 patients with a fixed follow-up of 5 years, we expect to see this number of events 4.7 years after the start of the study.

Supposing 50% of G1/2 patients are HR-CINSARC [9], 500 patients with interpretable CINSARC results are required. With 15% of screen-failure (non-interpretable CINSARC result) or random-failure, 600 patients need to be included.

An interim analysis for both futility (O Brien Fleming boundary) and efficacy (O Brien Fleming boundary) will be performed after observation of 51 events [18]. The East software package (v6.4) will be used to calculate the appropriate bounds at the time of the analysis, given the fraction of information available. An independent data monitoring committee will review the pre-planned interim analyses (futility and efficacy). The planned discontinuation boundaries and decision rules are as follows:

- 50% of expected events (51 events):
  - ◦ discontinuation for futility if one-sided *p*-value is > 0.282 (Hazard Ratio > 0.851)
  - ◦ discontinuation for efficacy if one-sided *p*-value is < 0.002 (Hazard Ratio < 0.438)
  - ◦ otherwise continue
- 100% of expected events (101 events):
  - ◦ claim success if one-sided *p*-value is < 0.024 (Hazard Ratio < 0.676)

The primary endpoint will be analyzed on the ITT population when the required number of events has been reached. The Kaplan-Meier approach will be used to estimate MFS rates for each treatment arm. Comparison between treatment arms will be performed using the Cox proportional hazards model with adjustment for stratification factors. Hazard ratios will be estimated with their 95% confidence interval (two-sided). The assumption of proportionality will be assessed graphically.

#### CINSARC prognostic value

In a retrospective study, the 3-year MFS was estimated to be 80 and 60% for LR and HR-CINSARC patients with FNLCC G1/2 [9, 10]. The main objective is to detect a hazard ratio of 2.1, which corresponds to a 3-year MFS equal to 80 and 63% in the LR and HR-CINSARC group, respectively. A total of 86 events is necessary for 90% power to detect this difference if it is true using a two–sided Logrank test at the 5% level of significance (it is assumed that 33% of G1/2 treated by standard of care are HR-CINSARC). Based on an estimated accrual rate of approximately 12 patients per month for the accrual of 375 patients treated by standard of care with a fixed follow-up of 5 years, we expect to see this number of events 4 years after the start of the study.

All patients FNCLCC G1/2 with interpretable CINSARC results will be treated according to standard of care. Survival rates (MFS, DFS and OS) will be estimated by CINSARC groups using the Kaplan-Meier method. Univariate analysis will be performed with the Logrank test. Variables associated with a univariate Logrank *p*-value less than 5% will be selected for multivariate analysis. Multivariate analysis will be performed with the Cox proportional hazards model to study the prognostic value of CINSARC groups on survival endpoints after adjusting for prognostic factors. Proportional hazards assumption will be assessed graphically.

## Discussion

This study protocol has several strengths. To our knowledge, this is the first randomized clinical trial to investigate the role of perioperative chemotherapy in STS patients G1/2 using a genomic signature to define HR patients. It has the capacity to evaluate treatment benefit in the HR-CINSARC population and to prospectively confirm the prognostic value of the CINSARC signature. However, this target selection design does not provide information regarding the lack of chemotherapy benefit in LR-CINSARC patients [19]. By using the CINSARC signature as a stratification criterion, about one half of the STS patients with G1/2 can be considered LR with a probability of less than 20% that the disease will recur. It seems reasonable to assume that the treatment benefit and the risk of toxicity is not well balanced in this subgroup, as many experts recommend chemotherapy only when the risk of metastatic disease is particularly high [20, 21].

Overall there is a strong rationale warranting a target selection design to validate the role of the CINSARC signature in G1/2 STS sarcoma [10, 13]. In the best-case scenario, this trial will confirm the value of perioperative chemotherapy in G1/2 STS presenting HR-CINSARC in grade ½ STS treated by standard of care. If the trial validates the prognostic value of the CINSARC signature in this patient population, it may easily be used to define eligibility criteria and stratification factors for future trials in G1/2 STS patients.

In summary, the rationale of the CHIC trial can be summarized as two interconnected goals:

- To determine the effectiveness of chemotherapy in grade ½ STS patients with HR-CINSARC;
- To prospectively validate the prognostic value of the CINSARC signature in grade ½ STS.

## Data Availability

Data sharing is not applicable to this article as no datasets were generated or analyzed during the current study.

## Abbreviations

STS: Soft tissue sarcoma
CINSARC: Complexity Index in SARComas
HR-CINSARC: High-Risk Complexity Index in SARComas
LR-CINSARC: Low-Risk Complexity Index in SARComas
G1/2: Grade 1/2
FFPE: Formalin-fixed paraffin-embedded
FNCLCC: Fédération Nationale des Centres de Lutte Contre le Cancer
CTCAE: Common Terminology Criteria for Adverse Events
MFS: Metastasis-free survival
DFS: Disease-free survival
OS: Overall survival

## References

[1] Adjuvant chemotherapy for localised resectable soft-tissue sarcoma of adults: meta-analysis of individual data. Sarcoma Meta-analysis Collaboration. Lancet. 1997;350:1647–54. https://pubmed.ncbi.nlm.nih.gov/9400508/. 9400508

[2] Pervaiz N, Colterjohn N, Farrokhyar F, Tozer R, Figueredo A, Ghert M (2008). A systematic meta-analysis of randomized controlled trials of adjuvant chemotherapy for localized resectable soft-tissue sarcoma. Cancer.

[3] Casali PG, Abecassis N, Aro HT, Bauer S, Biagini R, Bielack S, Bonvalot S, Boukovinas I, Bovee JVMG, Brodowicz T, Broto JM, Buonadonna A, De Álava E, Dei Tos AP, Del Muro XG, Dileo P, Eriksson M, Fedenko A, Ferraresi V, Ferrari A, Ferrari S, Frezza AM, Gasperoni S, Gelderblom H, Gil T, Grignani G, Gronchi A, Haas RL, Hassan B, Hohenberger P, Issels R, Joensuu H, Jones RL, Judson I, Jutte P, Kaal S, Kasper B, Kopeckova K, Krákorová DA, Le Cesne A, Lugowska I, Merimsky O, Montemurro M, Pantaleo MA, Piana R, Picci P, Piperno-Neumann S, Pousa AL, Reichardt P, Robinson MH, Rutkowski P, Safwat AA, Schöffski P, Sleijfer S, Stacchiotti S, Sundby Hall K, Unk M, Van Coevorden F, van der Graaf WTA, Whelan J, Wardelmann E, Zaikova O, Blay JY, ESMO Guidelines Committee and EURACAN (2018). Soft tissue and visceral sarcomas: ESMO-EURACAN Clinical Practice Guidelines for diagnosis, treatment and follow-up. Ann Oncol Off J Eur Soc Med Oncol.

[4] Benjamin RS (2017). Adjuvant and neoadjuvant chemotherapy for soft tissue sarcomas: a personal point of view. Tumori.

[5] Italiano A, Delva F, Mathoulin-Pelissier S, Le Cesne A, Bonvalot S, Terrier P, Trassard M, Michels J-J, Blay J-Y, Coindre J-M, Bui B (2010). Effect of adjuvant chemotherapy on survival in FNCLCC grade 3 soft tissue sarcomas: a multivariate analysis of the French Sarcoma Group Database. Ann Oncol Off J Eur Soc Med Oncol.

[6] Frustaci S, Gherlinzoni F, De Paoli A, Bonetti M, Azzarelli A, Comandone A, Olmi P, Buonadonna A, Pignatti G, Barbieri E, Apice G, Zmerly H, Serraino D, Picci P (2001). Adjuvant chemotherapy for adult soft tissue sarcomas of the extremities and girdles: results of the Italian randomized cooperative trial. J Clin Oncol Off J Am Soc Clin Oncol.

[7] Coindre JM, Terrier P, Bui NB, Bonichon F, Collin F, Le Doussal V, Mandard AM, Vilain MO, Jacquemier J, Duplay H, Sastre X, Barlier C, Henry-Amar M, Macé-Lesech J, Contesso G (1996). Prognostic factors in adult patients with locally controlled soft tissue sarcoma. A study of 546 patients from the French Federation of Cancer Centers Sarcoma Group. J Clin Oncol Off J Am Soc Clin Oncol.

[8] ESMO/European Sarcoma Network Working Group (2014). Soft tissue and visceral sarcomas: ESMO Clinical Practice Guidelines for diagnosis, treatment and follow-up. Ann Oncol Off J Eur Soc Med Oncol.

[9] Chibon F, Lagarde P, Salas S, Pérot G, Brouste V, Tirode F, Lucchesi C, de Reynies A, Kauffmann A, Bui B, Terrier P, Bonvalot S, Le Cesne A, Vince-Ranchère D, Blay J-Y, Collin F, Guillou L, Leroux A, Coindre J-M, Aurias A (2010). Validated prediction of clinical outcome in sarcomas and multiple types of cancer on the basis of a gene expression signature related to genome complexity. Nat Med.

[10] Valentin T, Lesluyes T, Le Guellec S, Chibon F (2019). Chemotherapy in localized soft tissue sarcoma: will we soon have to treat grade 1 tumors? Update on CINSARC performances. Ann Oncol Off J Eur Soc Med Oncol.

[11] Lesluyes T, Pérot G, Largeau MR, Brulard C, Lagarde P, Dapremont V, Lucchesi C, Neuville A, Terrier P, Vince-Ranchère D, Mendez-Lago M, Gut M, Gut I, Coindre J-M, Chibon F (2016). RNA sequencing validation of the complexity INdex in SARComas prognostic signature. Eur J Cancer Oxf Engl.

[12] Le Guellec S, Lesluyes T, Sarot E, Valle C, Filleron T, Rochaix P, Valentin T, Pérot G, Coindre J-M, Chibon F (2018). Validation of the complexity INdex in SARComas prognostic signature on formalin-fixed, paraffin-embedded, soft-tissue sarcomas. Ann Oncol Off J Eur Soc Med Oncol.

[13] Chibon F, Lesluyes T, Valentin T, Le Guellec S (2019). CINSARC signature as a prognostic marker for clinical outcome in sarcomas and beyond. Genes Chromosomes Cancer.

[14] Hoering A, Leblanc M, Crowley JJ (2008). Randomized phase III clinical trial designs for targeted agents. Clin Cancer Res Off J Am Assoc Cancer Res.

[15] Bellera CA, Penel N, Ouali M, Bonvalot S, Casali PG, Nielsen OS, Delannes M, Litière S, Bonnetain F, Dabakuyo TS, Benjamin RS, Blay J-Y, Bui BN, Collin F, Delaney TF, Duffaud F, Filleron T, Fiore M, Gelderblom H, George S, Grimer R, Grosclaude P, Gronchi A, Haas R, Hohenberger P, Issels R, Italiano A, Jooste V, Krarup-Hansen A, Le Péchoux C, Mussi C, Oberlin O, Patel S, Piperno-Neumann S, Raut C, Ray-Coquard I, Rutkowski P, Schuetze S, Sleijfer S, Stoeckle E, Van Glabbeke M, Woll P, Gourgou-Bourgade S, Mathoulin-Pélissier S (2015). Definition for the assessment of time-to-event endpoints in cancer trials initiative, guidelines for time-to-event end point definitions in sarcomas and gastrointestinal stromal tumors (GIST) trials: results of the DATECAN initiative (Definition for the Assessment of Time-to-event Endpoints in CANcer trials)†. Ann Oncol Off J Eur Soc Med Oncol.

[16] Geiss GK, Bumgarner RE, Birditt B, Dahl T, Dowidar N, Dunaway DL, Fell HP, Ferree S, George RD, Grogan T, James JJ, Maysuria M, Mitton JD, Oliveri P, Osborn JL, Peng T, Ratcliffe AL, Webster PJ, Davidson EH, Hood L, Dimitrov K (2008). Direct multiplexed measurement of gene expression with color-coded probe pairs. Nat Biotechnol.

[17] Malkov VA, Serikawa KA, Balantac N, Watters J, Geiss G, Mashadi-Hossein A, Fare T (2009). Multiplexed measurements of gene signatures in different analytes using the Nanostring nCounter Assay System. BMC Res Notes.

[18] Gordon Lan KK, DeMets DL (1983). Discrete sequential boundaries for clinical trials. Biometrika.

[19] Buyse M, Michiels S, Sargent DJ, Grothey A, Matheson A, de Gramont A (2011). Integrating biomarkers in clinical trials. Expert Rev Mol Diagn.

[20] Dangoor A, Seddon B, Gerrand C, Grimer R, Whelan J, Judson I (2016). UK guidelines for the management of soft tissue sarcomas. Clin Sarcoma Res.

[21] Gamboa AC, Gronchi A, Cardona K (2020). Soft-tissue sarcoma in adults: an update on the current state of histiotype-specific management in an era of personalized medicine. CA Cancer J Clin.

